# Cross-Cultural Validity, Reliability, and Psychometric Properties of the Persian Version of the Scales for Outcomes in Parkinson's Disease-Psychosocial Questionnaire 

**DOI:** 10.1155/2014/260684

**Published:** 2014-04-07

**Authors:** Seyed-Mohammad Fereshtehnejad, Farzaneh Farhadi, Hasti Hadizadeh, Gholam Ali Shahidi, Ahmad Delbari, Johan Lökk

**Affiliations:** ^1^Division of Clinical Geriatrics, Department of Neurobiology, Care Sciences, and Society (NVS), Karolinska Institutet, 14186 Stockholm, Sweden; ^2^Firoozgar Clinical Research Development Center (FCRDC), Firoozgar Hospital, Iran University of Medical Sciences, Tehran 15937-48711, Iran; ^3^Medical Student Research Committee (MSRC), Faculty of Medicine, Iran University of Medical Sciences, Tehran 14496-14535, Iran; ^4^Movement Disorders Clinic, Department of Neurology, Faculty of Medicine, Iran University of Medical Sciences, Tehran 14496-14535, Iran; ^5^Iranian Research Center on Aging, University of Social Welfare and Rehabilitation, Tehran 19857-13834, Iran; ^6^Department of Geriatric Medicine, Karolinska University Hospital, 14186 Stockholm, Sweden

## Abstract

*Objectives*. Considering the influence of different motor and nonmotor features of Parkinson's disease (PD), it is important to evaluate the psychosocial functioning of the patients. For this purpose, the scales for outcomes in Parkinson's disease-psychosocial questionnaire (SCOPA-PS) has been previously designed. The aim of our study was to assess the cross-cultural validation and psychometric properties of the Persian version of the SCOPA-PS. *Methods*. One hundred and ten nondemented idiopathic Parkinson's disease (IPD) patients were consecutively recruited from an outpatient referral movement disorder clinic. Eligible patients filled up a number of questionnaires including the Persian version of SCOPA-PS during the face-to-face interview session and clinical examination to measure disease severity, nonmotor psychiatric symptoms, and health-related quality of life (HRQoL). *Results*. The highest and lowest correlation coefficients of internal consistency were reported for item 7 on “*asking for help*” (*r* = 0.765) and item 5 on “*sexual problems*” (*r* = 0.553). Cronbach's alpha reliability coefficient of the entire scale was 0.87 (95% CI: 0.83–0.90). The Hoehn and Yahr stage (*r* = 0.34, *P* < 0.001), Schwab and England ADL scale (*r* = −0.55, *P* < 0.001), anxiety (*r* = 0.64, *P* < 0.001), depression (*r* = 0.71, *P* < 0.001), and fatigue (*r* = 0.35, *P* < 0.001) were significantly correlated with the total score of the SCOPA-PS questionnaire. *Conclusions*. The Persian version of SCOPA-PS is a highly reliable and valid scale to measure psychosocial functioning in IPD patients with different sex, age-group, and educational level, which could be applied in future researches. Disease severity scales, depression, anxiety, fatigue, and different domains of HRQoL were all associated with psychosocial functioning in PD patients.

## 1. Background

Parkinson's disease (PD) affects about 2% of population aged 65 years and over [1, 2]. In addition to motor symptoms, the disease also represents nonmotor features such as depression, apathy, mood changes, and increasing dependence on others for everyday activities [3]. As a result, it considerably influences the life and psychosocial functioning of PD patients; however, this consequence is rarely evaluated separately and in-depth [4, 5]. Psychosocial consequences of PD are commonly assessed by means of the health-related quality of life (HRQoL) instruments [6].

Since 5 billion of the world population live in the developing countries where life expectancy has been recently increased, these regions have to care for a larger number of patients with PD [2, 7]. While there are rare evidences on HRQoL and psychosocial aspects of PD available in the developing countries especially those of Asia and Africa, most studies have been performed in the developed countries [8–13].

Scales for outcomes in Parkinson's disease-psychosocial questionnaire (SCOPA-PS) is an 11-item instrument designed to evaluate psychosocial functioning and severity of mentioned problems in PD patients [14]. It has been validated in few countries and non-English languages including German, Spanish, and Brazilian [8, 14–16]. The aim of the present study was to perform an independent and cross-cultural validation of the Persian version of the SCOPA-PS. Moreover, we evaluated the psychometric properties of this questionnaire to show the relationships between motor and nonmotor symptoms of PD and psychosocial burden of the disease.

## 2. Methods

### 2.1. Study Setting

A total number of 110 idiopathic Parkinson's disease (IPD) patients who were consecutively admitted at an outpatient referral movement disorder clinic in Tehran, Iran, between October 2011 and September 2012 were included in the study. This study was a collaborative project between Karolinska Institute, Stockholm, Sweden, and Iran University of Medical Sciences (IUMS), Tehran, Iran. Using a cross-sectional design, eligible participants filled up the SCOPA-PS questionnaire during the face-to-face interview session and clinical examination was also performed together with assessments for psychiatric features, fatigue, and quality of life.

### 2.2. Ethical Considerations

The study protocol was approved by the Ethics Committee of the Neurology Department at Firoozgar Clinical Research Development Center (FCRDC) (affiliated to Iran University of Medical Sciences) in Tehran, Iran. The completion of the questionnaire was voluntary, and the aims and objectives of the study were explained to each patient before participation.

### 2.3. Sample and Procedure

Inclusion criteria were diagnosis of IPD based on the United Kingdom (UK) Brain Bank Criteria and age of 35 years or older [17]. Exclusion criteria were moderate-to-severe dementia with the Mini-Mental State Examination (MMSE) of <24 [18] and any patient with atypical Parkinsonian syndromes. After enrollment, a complete clinical examination was done by one neurologist specialized in movement disorders for all eligible participants.

Data collection was performed using four questionnaires including the SCOPA-PS, Parkinson's disease quality of life (PDQ-39), Hospital Anxiety and Depression Scale (HADS), and fatigue severity scale (FSS) to assess various aspects of the disease during the interview session and clinical examination. Demographic information consisted of baseline variables, educational status, and comorbidities. PD-related characteristics including disease duration (time passed from diagnosis), measures of disease severity such as Hoehn and Yahr stage [19], and Schwab and England activity of daily living (ADL) scale were recorded.

The Hoehn and Yahr staging [19] is a widely used clinical rating scale, which evaluates the severity of PD based on motor functional disability and clinical findings consisting of 5 stages. Stage 0 indicates no visible symptoms of PD, and stage 5 shows symptoms on both sides of the body indicating the PD patients who are unable to walk. Therefore, a higher stage shows greater levels of functional disability [19]. The Schwab and England scale is another global scoring system for assessing a PD patient's ability to perform daily activities in terms of speed and independence through a percentage figure, where 100% indicates total independence and 0% indicates a state of complete dependence in bed-ridden individuals. Therefore, higher scores show greater level of independence. Moreover, the validated Persian version of the fatigue severity scale (FSS-Per) [20] was used to measure fatigue in recruited PD patients. It contains nine questions asking participants to rate the level of fatigue during the past week, while rating scores range from 1 to 7 for each statement. A total score is obtained as the average of all the item-specific scores where higher scores show more severe fatigue [21]. As a screening tool designed to assess the levels of anxiety and depression in patients attending medical clinics, the Persian version of the Hospital Anxiety and Depression Scale (HADS) was also used [22]. It consists of 14 questions divided into two sections; seven questions are related to anxiety and the other seven questions focus on depression. Each section is worth 0–21 points, providing separate scores for either anxiety or depression where the higher scores demonstrate worse condition [23]. As the most common instrument to measure health-related quality of life in PD patients, we also used Parkinson's disease quality of life questionnaire (PDQ-39) [24]. It contains 39 items assessing eight domains of quality of life (QoL) in PD patients: mobility, activities of daily living (ADL), emotional well-being, stigma, social support, cognitions, communication, and bodily discomfort. All questions in PDQ-39 are coded in a Likert scale from 0 to 4, where 0 = never, 1 = occasionally, 2 = sometimes, 3 = often, and 4 = always. The maximum score of 100 on the PDQ-39 scale represents the worst conditions, while a zero score represents the best condition of QoL in PD patients [24]. In this study, we used a Persian-translated version of the PDQ-39 questionnaire, which has been previously validated [25]. All the assessments were done when the patients were in the “On” state.

### 2.4. SCOPA-PS Questionnaire

The SCOPA-PS questionnaire [14] is a self-administered 11-item scale assessing psychosocial functioning and the severity of a particular problem during the last month. Items are scored with a four-point Likert scale, from 0 (not at all) to 3 (very much). By adding the scores on the individual items, the sum score is calculated, and by transforming the mentioned sum score into percentage values (on the maximum possible score, 33 points), summary index (SCOPA-PS SI) is computed. The higher the summary index is, the worse HRQoL is expected in psychosocial aspects [14].

### 2.5. Translation and Back-Translation

Three native Persian speakers fluent in English translated the SCOPA-PS into Persian. Later on, an English native fluent in Persian who was not involved in the study back-translated the SCOPA-PS into English with no access to the original version of the questionnaire. In the comparison of the first back-translation with the original version, modifications were made to eliminate all discrepancies. The final joint translation was named SCOPA-PS, Persian version.

### 2.6. Statistical Analysis

#### 2.6.1. Description

Data were analyzed by SPSS software version 17.0 (Chicago, IL, USA). To describe continuous variables, mean [standard deviation (SD)] was calculated and frequency (percentage) was used for nominal and categorical variables. The minimum, maximum, and coefficient of variation (CV) were also reported for each of the items in SCOPA-PS questionnaire. In order to guarantee the acceptability of a scale, floor and ceiling effects were calculated, which should be less than 15% [26].

#### 2.6.2. Reliability

In reliability analysis, internal consistency was assessed using Spearman correlation statistic where the mean score of each item was correlated with the sum of the SCOPA-PS score in Parkinsonian patients. Furthermore, Cronbach's alpha intraclass coefficient and the 95% confidence interval (CI) of the point estimations were also calculated for the whole questionnaire and within different subgroups of IPD patients regarding age-group, sex, and level of education.

#### 2.6.3. Validity

Spearman correlation was used to evaluate the convergent validity of the total score of the SCOPA-PS questionnaire in association with demographics and PD-related variables.

In all analytical procedures, a two-sided *P* value <0.05 was considered as the statistical significant level to reject the beyond H0 hypothesis.

## 3. Results

### 3.1. Sociodemographic Characteristics

The study population consisted of 34 (30.9%) females and 76 (69.1%) males with the mean age of 61.61 (SD = 10.97) years. Sociodemographic characteristics of the recruited participants are summarized in Table 1. The mean duration of PD was 6.34 (SD = 4.74) years and the median stage of disease severity was 2 based on the Hoehn and Yahr grading. Depression (22.7%, *n* = 25) and hypertension (16.4%, *n* = 18) were reported to be the most common comorbidities in PD patients, respectively.

### 3.2. SCOPA-PS Data Description

The Persian version of the SCOPA-PS was filled up by 110 PD patients with no missing value for any of the 11 items.  Table 2 describes the distribution of the answers for each question and their mean scores. The highest score was observed in item 11 on “*future concerns*” with the mean of 1.42 (SD = 1.10), whereas the lowest score was recorded for item 4 on “significant others” with the mean of 0.38 (SD = 0.78). The mean relative score of the whole questionnaire was 25.23% (SD = 22.20) ranging between 0 and 97%. SCOPA-PS had no floor effect, and the ceiling effect was 14.5%.

### 3.3. Reliability

As listed in Table 2, all of the items showed significant correlation to the total score of the SCOPA-PS questionnaire (all *P* < 0.001). The highest and lowest Spearman correlation coefficients were reported for item 7 on “*asking for help*” (Rho = 0.765) and item 5 on “*sexual problems*” (Rho = 0.553). Cronbach's alpha coefficient was also calculated for the assessment of the internal consistency reliability of the questionnaire, which resulted in 0.87 (95% CI: 0.83–0.90). Deletion of none of the questions would increase Cronbach's alpha coefficient above the formerly calculated overall coefficient (0.869). The highest single-item sensitivity of Cronbach's alpha reliability was reported for item 8 on “*loneliness*” and item 1 on “*difficulty at work*” where the coefficient would decrease to as low as 0.850 if either of these two questions was deleted.

Cronbach's alpha reliability coefficient was also calculated within different subgroups of PD patients with regard to age-group, sex, and educational level. As shown in Table 3, the Persian version of the SCOPA-PS questionnaire had higher Cronbach's alpha coefficient in males [0.89 (95% CI: 0.85–0.92) versus 0.80 (95% CI: 0.68–0.89)] and less educated participants [0.90 (95% CI: 0.85–0.94) versus 0.83 (95% CI: 0.76–0.88)].

### 3.4. Validity

**Figure 1:**
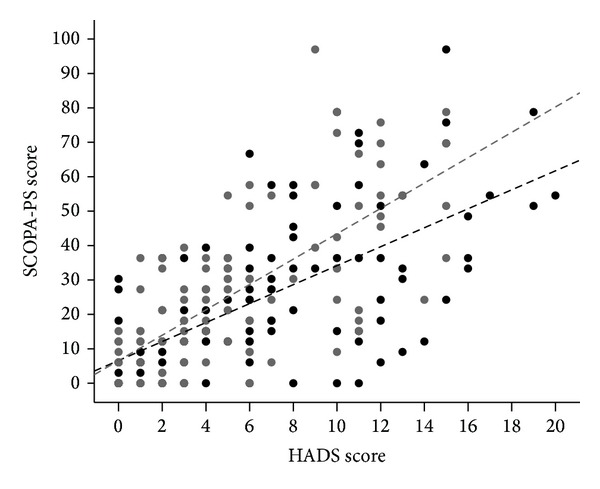
Scatter plot of the correlation between total score of SCOPA-PS and HADS questionnaires for anxiety (black circles; Spearman Rho = 0.642, *P* < 0.001) and depression (gray circles, Spearman Rho = 0.715, *P* < 0.001) in Iranian Parkinson's disease patients (*n* = 110).

Higher Hoehn and Yahr stage (Spearman Rho = 0.34, *P* < 0.001) and lower Schwab and England ADL scale (Spearman Rho = −0.55, *P* < 0.001) were significantly correlated with the total score of the SCOPA-PS questionnaire (Table 4). All of the domains of PDQ-39 were also directly correlated with the SCOPA-PS score and the highest correlation coefficient was recorded in the mobility domain (Spearman Rho = 0.72, *P* < 0.001). The entire score of the PDQ-39 was strongly correlated with the SCOPA-PS score as well (Spearman Rho = 0.82, *P* < 0.001). Moreover, anxiety (Spearman Rho = 0.64, *P* < 0.001), depression (Spearman Rho = 0.71, *P* < 0.001), and fatigue (Spearman Rho = 0.35, *P* < 0.001) were all correlated with the total score of the SCOPA-PS questionnaire.  Figure 1 illustrates the scatter plot between the total SCOPA-PS score and HADS scores of anxiety and depression.

## 4. Discussion

Until now, SCOPA-PS has been translated into Latin-American and few European languages [15, 16], yet no Persian-translated version of SCOPA-PS is available in Iran and the present study is the first attempt in validating this version of SCOPA-PS. Indeed, this effort is of utmost importance to validate an instrument to measure psychosocial burden of PD in patient population.

The internal consistency of SCOPA-PS was acceptable (Cronbach's alpha = 0.87) in our study and was even higher than the original study and the Brazilian version (Cronbach's alpha = 0.83) [14, 16] where the intraclass correlation was also high. Results of similar studies on validity and reliability of the SCOPA-PS in different languages are summarized in Table 5. Originally, Marinus et al. developed the SCOPA-PS to evaluate psychosocial functioning in PD patients. One hundred and seventeen PD patients filled the Dutch version of the SCOPA-PS in stage 1 to 5 of disease severity according to Hoehn and Yahr. The result revealed that the Dutch version of SCOPA-PS had good internal consistency (Cronbach's alpha = 0.83) [14].

In item-specific analysis, the highest correlation score (0.76) referred to item 7 on “*asking for help*” which indicates the highest internal consistency with the total SCOPA-PS score. Even though all coefficients were above 0.4 as the limit for item consistency, the lowest coefficient (0.55) was observed for item 5 on “*sexuality.*” Moreover, deletion of none of the questions would increase the total Cronbach's alpha coefficient above the formerly calculated one, which means that all of the questions are suitable for the internal consistency. However, cultural barriers about sexual aspects and taboo considerations may cause the relatively low internal consistency of item 5 on psychosocial problems with “*sexuality.*” In line with our findings, the lowest consistency coefficient in both the original Dutch [14] and the German versions [15] of the questionnaire was also reported for item 5 on “*sexuality*”, whereas in the Brazilian [16] and Spanish versions [8] item 10 on “*feeling ashamed of disease*” showed the lowest consistency. Interestingly, the coefficient for the “sexuality” item was found to be higher in our study compared to the reports from the Netherlands [14] and Germany [15], while the opposite might have been expected according to the sociocultural characteristics. This may highlight the evolutionary transition of current Iranian society, showing less taboo talking about sexual issues especially in the group of patients with chronic disabilities. At the same time, it could also indicate the importance of sexual problems in the functioning of Parkinsonian patients that makes them precisely answer the question on sexuality. This finding was actually against our expectations prior to data collection to encounter problems in the validity and reliability of the “sexuality” item.

According to our results, patients with higher Hoehn and Yahr stage and lower Schwab and England ADL scale had higher SCOPA-PS score showing the fact that patients with more severe PD have more problems with their psychosocial functioning. Different psychiatric features including anxiety (*r* = 0.64), depression (*r* = 0.72), fatigue (*r* = 0.36), and all of the subscales and entire HRQoL measured by the PDQ-39 were also correlated with the total score of SCOPA-PS. These findings not only further confirm the validity of the Persian version of the SCOPA-PS questionnaire, but also show the important relationships between different symptoms (both motor and nonmotor) of PD and psychosocial malfunctioning in patients. Previously, the Brazilian study of SCOPA-PS also showed significant correlation between total score of SCOPA-PS and anxiety (*r* = 0.51), depression (*r* = 0.49), and Hoehn and Yahr stage (*r* = 0.38) [16]. Likewise, in the original study that developed the SCOPA-PS with a sample size of 338 PD patients, total score of the questionnaire was significantly correlated with PDQ-39 score (*r* = 0.82), anxiety (*r* = 0.62), and depression (*r* = 0.61) [14].

As one strength point, we used several scales and questionnaires to consider PD severity and different motor, nonmotor, and psychiatric features in relation with the psychosocial functioning of PD patients. Therefore, it was possible to evaluate different relationships and interactions in cross-cultural validation of the Persian version of SCOPA-PS. However, this study was designed as a cross-sectional study, which made it impossible to evaluate test-retest reliability, and any interpretation on causal relationships between PD-related symptoms and psychosocial functioning could not be made. Another limitation is based on the fact that the present study was performed in an outpatient clinic that mostly included mild-to-moderate PD patients, which makes it difficult to generalize our findings to the end-stage and hospitalized patients.

## 5. Conclusions

In conclusion, our study demonstrated that the Persian version of SCOPA-PS is a reliable and valid scale to measure psychosocial functioning in IPD patients with different sex, age-group, and educational level. This cross-culturally validated version of SCOPA-PS has high reliability for individual assessment. Opposite to our prestudy assumptions, Iranian PD patients precisely answered the SCOPA-PS questionnaire and less cross-cultural validation was needed to adopt the items of this questionnaire with the sociodemographic contexts of our society.

During the process of convergent validity, PD severity scales, depression, anxiety, fatigue, and different domains of HRQoL were all associated with psychosocial functioning measured by the SCOPA-PS. Comparing with results from other similar studies, the item-specific analysis confirmed the importance of cross-cultural validation for psychological scales; for instance, in some cultures, the answers to questions about sexuality are less valid. In total, our investigation recommends the Persian version of the SCOPA-PS questionnaire as a valid and reliable tool to measure psychosocial functioning in PD patients, which could be used in future projects.

## Figures and Tables

**Table 1:** Sociodemographic characteristics of Parkinson's disease patients (*n* = 110).

Characteristics	Value
Age (yr) Mean (SD)	61.6 (11.0)
Gender number (%)	
Female	34 (31)
Male	76 (69)
Level of education number (%)	
Illiterate	8 (7)
Primary and/or secondary	28 (26)
High school/diploma	30 (28)
College and/or university	43 (39)
Duration of disease (yr) Mean (SD)	6.3 (4.7)
Comorbidities number (%)	
Depression	25 (23)
Hypertension	18 (16)
Cardiovascular disease	17 (16)
Osteoarthritis	12 (11)
Diabetes	11 (10)
Hoehn and Yahr stage Mean (SD)	1.9 (0.9)
Schwab and England activities of daily living scale (%) Mean (SD)	82.0 (16.5)

**Table 2:** Data distribution, description, and Spearman correlation for internal consistency of the SCOPA-PS scores in Iranian Parkinson's disease patients (*n* = 110).

Item	Answer distribution number (%)				Descriptive indexes		Spearman Rho*
	Not at all (0)	A little (1)	Quite a bit (2)	Very much (3)	Mean	SD	
Item 1	68%	12%	12%	8%	0.60	0.99	0.656
Work							
Item 2	65%	11%	18%	6%	0.66	0.99	0.623
Hobbies							
Item 3	73%	9%	14%	4%	0.47	0.86	0.601
Interacting with others							
Item 4	78%	7%	13%	2%	0.38	0.78	0.596
Significant others							
Item 5	64%	12%	16%	8%	0.69	1.02	0.553
Sexuality							
Item 6	56%	10%	19%	15%	0.95	1.17	0.703
Staying at home							
Item 7	52%	10%	22%	16%	1.03	1.18	0.765
Help							
Item 8	58%	16%	19%	7%	0.75	1.01	0.740
Lonely							
Item 9	67%	13%	17%	3%	0.55	0.87	0.588
Conversation							
Item 10	57%	15%	17%	11%	0.82	1.08	0.578
Embarrassment							
Item 11	29%	19%	33%	19%	1.42	1.10	0.565
Future							
Total score (crude)	—	—	—	—	**8.33**	**7.33**	—
Total score (relative %)	—	—	—	—	**25.23**	**22.20**	—

*All correlation coefficients are statistically significant with *P* < 0.001.

**Table 3:** Reliability (Cronbach's *α*) of the SCOPA-PS questionnaire within various subgroups of Iranian Parkinson's disease patients (*n* = 110).

Subgroups	Cronbach's *α* coefficient (95% CI)
Total (*n* = 110)	**0.87 (0.83 to 0.90)**
Age-group	
<65 yr (*n* = 65)	0.88 (0.84 to 0.92)
≥65 yr (*n* = 45)	0.84 (0.76 to 0.90)
Gender	
Female (*n* = 34)	0.80 (0.68 to 0.89)
Male (*n* = 76)	0.89 (0.85 to 0.92)
Educational level	
Illiterate/primary/secondary (*n* = 36)	0.91 (0.85 to 0.95)
College/university (*n* = 73)	0.83 (0.76 to 0.88)

**Table 4:** Spearman correlation for convergent validity of the SCOPA-PS questionnaire regarding other measured scales and variables in Iranian Parkinson's disease patients (*n* = 110).

Scale/variable	Spearman Rho
Age	0.07
Duration of disease	0.17
Hoehn and Yahr stage	0.34*
Schwab and England ADL scale (%)	−0.55*
PDQ-39 (quality of life)	
Mobility	0.72*
Activities of daily living (ADL)	0.60*
Emotional well-being	0.63*
Stigma	0.46*
Social support	0.43*
Cognitive impairment (cognition)	0.54*
Communication	0.54*
Bodily discomfort	0.44*
Total	0.82*
HADS	
Anxiety	0.64*
Depression	0.72*
FSS (fatigue)	0.36*

*Statistical significant correlation at the level of *P* < 0.001.

**Table 5:** Validity, reliability, and psychometric properties of the SCOPA-PS in different languages/cultures (two different coefficients are reported for some indices to show the lowest and highest item-specific calculated reliability).

Authors	Year	Country/language	Reliability		Validity		
			Internal consistency (Spearman R)	Internal consistency (Cronbach's alpha)	Correlation coefficient with HADS (anxiety)	Correlation coefficient with HADS (depression)	Correlation coefficient with HRQoL (PDQ-39)*
Marinus et al. [14]	2003	The Netherlands/Dutch	0.24 (item 5) 0.67 (item 7)	0.83	0.61	0.62	0.82

Carod-Artal et al. [16]	2006	Brazil/Brazilian and Portuguese	0.43 (item 10) 0.73 (item 9)	0.84	0.50	0.47	0.73

Virués-Ortega et al. [8]	2009	Argentina, Paraguay, Ecuador, Brazil/Spanish and Portuguese	0.43 (item 10) 0.71 (item 9)	0.87	0.62	0.61	0.82

Knudsen et al. [15]	2007	Germany/German	0.35 (item 5) 0.77 (item 6)	0.90	0.76	0.76	0.86

Current study	2013	Iran/Persian	0.55 (item 5) 0.76 (item 7)	0.87	0.64	0.71	0.82

*The reported correlation coefficients refer to the total score of the PDQ-39 questionnaire.
